# Regulatory inspection of registered private drug shops in East-Central Uganda—what it is versus what it should be: a qualitative study

**DOI:** 10.1186/s40545-020-00265-9

**Published:** 2020-09-10

**Authors:** Arthur Bagonza, Stefan Peterson, Andreas Mårtensson, Henry Wamani, Phyllis Awor, Milton Mutto, David Musoke, Linda Gibson, Freddy Eric Kitutu

**Affiliations:** 1 Department of Community Health and Behavioural Sciences, School of Public Health, Makerere University College of Health Sciences, Kampala, Uganda; 2 Department of Health Policy Planning and Management, School of Public Health, Makerere University College of Health Sciences, Kampala, Uganda; 3 International Maternal and Child Health Unit, Department of Women’s and Children’s Health, Uppsala University, Uppsala, Sweden; 4 Department of Disease Control and Environmental Health, School of Public Health, Makerere University College of Health Sciences, Kampala, Uganda; 5 School of Social Sciences, Nottingham Trent University, Nottingham, UK; 6 Department of Pharmacy, School of Health Sciences, Makerere University College of Health Sciences, Kampala, Uganda

**Keywords:** Drug sellers, Drug shops, Inspection, Regulation, Supervision, Uganda

## Abstract

**Background:**

Regulatory inspection of private drug shops in Uganda is a mandate of the Ministry of Health carried out by the National Drug Authority. This study evaluated how this mandate is being carried out at national, district, and drug shop levels. Specifically, perspectives on how the inspection is done, who does it, and challenges faced were sought from inspectors and drug sellers.

**Methods:**

Six key informant interviews (KIIs) were held with inspectors at the national and district level, while eight focus group discussions (FGDs) were conducted among nursing assistants, and two FGDs were held with nurses. The study appraised current methods of inspecting drug sellers against national professional guidelines for licensing and renewal of class C drug shops in Uganda. Transcripts were managed using Atlas ti version 7 (ATLAS.ti GmbH, Berlin) data management software where the thematic content analysis was done.

**Results:**

Five themes emerged from the study: authoritarian inspection, delegated inspection, licensing, training, and bribes. Under authoritarian inspection, drug sellers decried the high handedness used by inspectors when found with expired or no license at all. For delegated inspection, drug sellers said that sometimes, inspectors send health assistants and sub-county chiefs for inspection visits. This cadre of people is not recognized by law as inspectors. Inspectors trained drug sellers on how to organize their drug shops better and how to use new technologies such as rapid diagnostic tests (RDTs) in diagnosing malaria. Bribes were talked about mostly by nursing assistants who purported that inspectors were not interested in inspection per se but collecting illicit payments from them. Inspectors said that the facilitation they received from the central government were inadequate for a routine inspection.

**Conclusion:**

The current method of inspecting drug sellers is harsh and instills fear among drug sellers. There is a need to establish a well-recognized structure of inspection as well as establish channels of dialogue between inspectors and drug sellers if meaningful compliance is to be achieved. The government also needs to enhance both human and financial resources if meaningful inspection of drug sellers is to take place.

## Introduction

Regulation of private drug sellers remains a global challenge which has contributed to inappropriate treatment ultimately resulting in drug resistance because of policy and implementation challenges [1, 2]. Such challenges include but may not be limited to social, cultural, organization, and contextual ones [3]. In many healthcare settings, inspection is done by external personnel who make sure that laws and standards are adhered to by practitioners [4]. In public health care settings, inspection is offered in an orderly and well-structured manner with clear audit-and-feedback channels which have proved to complement training leading to desired treatment outcomes [5]. On the contrary, evidence reveals great uncertainty of whether inspection of private for-profit healthcare providers in low- and middle-income countries improves quality of care [6, 7]. This may be due to inspection visits being brief, unstructured, punitive, and not aimed at problem-solving [8]. This has been attributed to a shortage of trained human resources for inspection, inadequate financing, and under-qualified inspectors [9, 10].

In Sub-Saharan Africa where about 60% of the people live in rural areas [11], the healthcare needs of the vast majority are served by small drug shops devoid of big pharmacies and only allowed to sell over-the-counter drugs (OTC) belonging to a particular class [12, 13]. In many Sub-Saharan countries, policies are prescriptive on who should operate private pharmacies and drug shops. However, quite often, the people licensed are not the ones that operate the pharmacies or small drug shops [14, 15] which further justifies the need for inspection. To further illuminate this evidence, research shows that because of the weak inspection, the misuse of drugs, and inappropriate treatment of patients in the private sector continues unabated [13, 16].

In order to understand the social realities underpinning inspection aimed at achieving compliance, the regulation theory using the wheel of social alignment was used [17]. The theory pays special attention to the importance of the psycho-social determinants of compliance such as shame, trust, identity, norms, guilt, and values. The theory also highlights the need for regulators to understand the legislation of inspection, have guidance for interpretation of the inspection policy, and understand the intent of the policy. More so, regulators need to be aware of the best ways to enforce the practice of the people being inspected and be cognisant of the investigative rules. From the perspective of those being inspected, the theory interrogates the primary factors shaping compliance and what is being asked from them. Such may include benefits that may accrue from being inspected, whether justice will prevail, and whether they feel any moral obligation to be inspected.

In Uganda, more than 50% of people in rural areas get their first form of ambulatory care from private for-profit healthcare providers mainly made up of drug shops [18]. However, statutory policy prescribes that a large number of drug shops in a district shall be inspected by one person qualified as a pharmacy technician [19]. This often leads to work overload and inefficient inspection ultimately resulting in drug sellers maximizing profit through the inappropriate prescription of drugs especially antibiotics to patients [20, 21]. Moreover, while the inspection policy for drug sellers in Uganda is prescriptive in as far as who the inspectors should be and what they should do, it is not clear whether what is prescribed is what actually happens during the inspection. It was envisaged that there may be a difference between policy and practice as far as inspection of drug sellers in Uganda is concerned.

### Aim

This study appraised the current methods of inspecting drug sellers against national professional guidelines for licensing, renewal, and new licenses for class C drug shops in Uganda. More specifically, the study explored perspectives of drug sellers and their inspectors regarding methods of inspecting drug shops.

## Methods

### Study design

This was a descriptive qualitative study which employed key informant interviews (KIIs) and focus group discussions (FGDs). KIIs and FGDs have been recommended as methods of eliciting information in qualitative descriptive approaches [22, 23]. A qualitative inquiry methodological approach was used by investigators of the study because they wanted a comprehensive analysis of regulatory inspection as it occurs in Uganda [24]. The researchers also chose to use a qualitative descriptive study because they wanted to stay closer to the data and surface of the gathered information, words, and events as prescribed by Sandelowski [24, 25].

### Study setting and participants

This study was conducted in Luuka and Buyende districts, two rural districts in East-Central Uganda. Luuka District has an active drug shop association which has a committee headed by a chairperson. The association organizes monthly meetings where drug sellers meet to deliberate on matters concerning licenses and daily running of drug shop operations. On the other hand, Buyende district does not have a drug shop association. The projected population of children under 5 years by December 2020 is 88,860 in Buyende district and 48,200 in Luuka District respectively [26]. Both districts have no hospital while each district has 1 Health Centre (HC) IV, several HC IIIs, and HC IIs. The majority of the population seeks the first form of treatment from drug shops which are found in all villages.

For key informant interviews, we interviewed inspectors at national and district levels. At the national level, we interviewed an official from the Ministry of Health (MoH) and another from the National Drug Authority (NDA) in charge of inspection of pharmacies and drug shops in Uganda. At the district level, we interviewed District Health Officers (DHOs) and District Drug Inspectors (DDIs).

In the focus group discussions which were conducted at the district level, only registered drug sellers were interviewed. Identifiers that may have been used to disclose the people interviewed in this study were removed in line with protecting the confidentiality of respondents in qualitative studies [27].

### Methods of data collection

We purposively sampled participants for this qualitative study because they were well informed about the regulation of drug shops in Uganda [28]. The sampling was based on (1) being a statutorily recognized inspector and (2) being a licensed drug seller.

#### Key informant interviews

Six inspectors (four at district and two at the national level) were purposively sampled for key informant interviews (KIIs). The KIIs were conducted from the offices of the respondents. During interviews, where necessary, probing questions followed the main questions to get a deeper understanding of the participants’ views. This was done until subsequent interviews yielded no new information to warrant additional interviews [29]. The interview guide explored how inspection of registered drug sellers is done, who does the inspection, challenges experienced during the inspection process, and how these challenges may be overcome. Saturation was achieved by the fifth KII. The team leader (AB) informed the key informants about the interviews both verbally and in writing.

#### Focus group discussions

In each district, one FGD was conducted with nurses while eight FGDs comprised of ten participants per FGD participated in the study in both districts. In total, one hundred licensed drug sellers were purposively selected for focus group discussions (FGDs). Two of the FGDs (one from either district) comprised of midwives, comprehensive nurses and enrolled nurses (collectively referred to as nurses for purposes of this study), while the rest of the eight FGDs comprised of nursing assistants. Each FGD is comprised of ten participants which are desirable for an FGD [30]. The FGDs were conducted from the sub-county headquarters in each district at places devoid of noise. The team leader (AB) informed the drug sellers about the interviews through the DHOs, while the drug sellers were mobilized by the DDIs who were informed by the DHOs.

### Training and quality control

Three research assistants (RAs) with degrees in social sciences from Makerere University were recruited and given refresher training on qualitative data collection methods and tools. One of the two RAs were engaged in interviewing key informants while the other RA moderated FGDs. The third RA took notes during FGDs while the study leader (AB) took notes during KIIs. Each KII took an average of 50 min to complete while each FGD took an average of 65 min. The interview guide was in English but moderators conversant with English and Lusoga—the native dialect in Luuka and Buyende districts were used in conducting the KIIs and FGDs. Lunch was provided to all participants since all interviews started at 11 am every morning. This time was agreed upon to enable drug sellers from villages far away from the sub-county headquarters to join drug sellers who hailed from nearby areas. All discussions and interviews were audio-recorded.

### Data management and analysis

The transcription of all the interviews was done over a 2-week period and transcripts were uploaded into Atlas ti version 7—a qualitative data management software (ATLAS.ti GmbH, Berlin). The primary researcher, who was a PhD student, assumed an interpretivist ontological and constructivist epistemological position because he believed that the reality of regulatory inspection in Uganda had multiple facets, is relative and that the experience of being inspected is socially constructed [31–33]. That is to say, besides experiences of being inspected being influenced by the individual’s academic qualifications and being statutorily licensed, the lived experiences are socially constructed. The data was analyzed with the aim of understanding the current approach of regulatory inspection in Uganda in order to recommend improvements where the approach fell short of ideal standards. Text from uploaded KIIs and FGDs transcripts were selectively divided into meaning units and subsequently into condensed meaning units [34]. Taking context into consideration, the condensed meaning units were abstracted and labeled with codes by AB, and the research assistants generated over 40 codes from the first two KII and FGD transcripts. These codes were compared for similarities and differences before they were merged. A comparison of codes was done collaboratively with PhD academic supervisors and compared with codes generated by MM (another PhD student with qualitative proficiency but not connected to the study). The agreed-upon codes together with other emerging ones were then used to code the rest of the transcripts which increased the trustworthiness of the results [35, 36]. The use of different personnel in the triangulation process is believed to have improved the interpretive rigor of the study [35, 37]. The various codes were then compared for similarities and differences before being sorted and merged to form sub-themes. The sub-themes were then compared and condensed into higher-order themes. Emerging themes were agreed upon by the researchers of this study and compared with themes suggested by MM until consensus was reached. During analysis, a thematic analysis using template analysis was used for this study. Templates are used when researchers have codes a priori but also allow the generation of new codes when information collected from subsequent interviews does not fall within already pre-generated codes [38, 39]. The codes then aided the formation of sub-themes and overall themes after a series of condensation. The template that guided thematic analysis for this study was constructed using codes generated from interviews with participants of the study [40, 41]. The themes were then compared with the national policy for inspection of drug shops in Uganda.

## Results

Five broad themes emerged from the data as follows: authoritarian inspection, passive inspection, licensing, training, and bribes. Our results show that the regulation of drug shops is mainly about licensing and inspection. The inspection is unpredictable, authoritarian, and characterized by illicit payments whenever it happens. Drug sellers felt that if the licensing process was made easier coupled with more training courses on childhood diseases, this would contribute to smooth running of the business. Drug sellers recommended that support supervision should be initiated to augment inspection.

The mean (SD) age of the inspectors was 47 (5.1) years and has spent an average (SD) of 7(2) years in service. One hundred drug sellers participated in FGDs and were divided into nurses and nursing assistants. There were 20 nurses whose average (SD) age was 33 (8) years and had spent 8(4.8) years as drug sellers. The mean (SD) age of the nursing assistants was 32(7) years and had spent 7(6) years in service.

### Authoritarian inspection

All the inspectors at the national level described inspection as a process that involves making sure that drug sellers adhere to policy guidelines. They described the process as seeking clearance to open premises that would be used as drug shops from the office of the DHO. Once triggered, this process would begin by inspecting premises, the surrounding environment, as well as ascertaining the qualifications of the drug sellers. On fulfilling these conditions, a formal application process leading to licensing was initiated. Thus, during a routine inspection, premises, personnel, drugs being sold and the environment were the major areas of focus.

“What I know, usually for inspection, first, for you to open, you need clearance from the DHO. In the office of the DHO used to be someone called the District Drug Inspector. This person was being financially supported by the National Drug Authority. So what would happen, the DDI would go and see the premises and would be the one to open if satisfied that the premises meet the required parameters and the staffing.” (KII, Inspector, MoH)

As far as nurses were concerned, the focus group in Luuka District said that the current method of the inspection was more to do with adherence to policy guidelines. The majority of members in the focus group said that when inspectors went to drug shops and found them disorganized, drug sellers were advised on what to do. When the inspectors returned and found the drug sellers had adjusted accordingly, they praised and encouraged them to keep it up. However, when inspectors returned and nothing about the drug shop had changed, the drug sellers were apprehended. This was one of the positive findings noted about the current method of inspection.

On the contrary, the focus group discussion of nurses in Buyende district said that inspectors were high handed if they found drug sellers with an expired license or without one at all. This, they said, led to automatic confiscation of drugs regardless of the circumstances.

“When they find you without a license they just take all your drugs. Sometimes you have just secured a loan, then you get a setback and start afresh because they have taken each and everything.” (FGD, Nurses, Buyende district)

All focus group discussions with nursing assistants in both districts revealed that the current method of the inspection was impromptu, did not give drug sellers ample time to put things in order, and involved the use of law enforcement personnel who evoked fear among drug sellers. This was said in light of regional inspectors from the NDA who carried out inspection accompanied by police every time inspection visits were carried out. The regional inspectors stormed into drug shops and demanded to see the qualifications of the seller. Drug sellers said that the inspectors did not give them a chance to show their qualifications even if they had kept them away from their shops. The requirement was that every drug seller was supposed to hang their license and have their academic qualifications nearby. Short of that, drug sellers were harassed and in some cases, arrested. In particular, nursing assistants said that during the inspection, the main intention was to confiscate drugs that may not be legally sold by drug sellers as well as embarrass and apprehend perpetrators. Drug sellers argued that there was a need to improve the manner in which inspection was done by reducing harshness.

“Yes, when they reach they are very harsh, they have a very unpleasing language. They embarrass us and say that we know nothing. That really hurts us.” (FGD, Nursing assistants, Luuka District)

However, inspectors attributed the fear among drug sellers to opening and trading illegally. They said that drug sellers had no reason to fear if they had been vetted and were properly licensed. Also, inspectors said that they rarely informed drug sellers when they were going for inspection visits because most of them closed their drug shops since many lacked licenses which are a pre-requisite for operation.

“Sometimes, drug inspectors want to access the drug shops but there is fear because most of them are operating illegally. When you go to a certain trading center, only 2 or 3 drug sellers can welcome you. So they don't give us a chance to inspect them properly.” (KII, Inspector, Luuka district)

### Delegated inspection

All KIIs with inspectors at the district level revealed that during district monthly meetings, DDIs gave an update on the status of drug shops in their jurisdiction. However, a fully detailed inspection report was prepared and shared with district authorities every quarter. It was also noted that whereas the DDIs were the main people involved in the inspection, other members of the district health team such as the district health visitor and assistant DHO in charge of the environment were also involved in the inspection of drug shops. The inspection of drug shops was integrated with activities of other members of the district health team to ensure inspection takes place even when funds from the central government delay. The inspectors said that they have a large geographical area with drug shops to inspect and as such, the DDIs of each district inspect a different sub-county every month in no particular order. Even then, this depended on the availability of funds to carry out inspection

“We make quarterly reports to districts and monthly district health team meetings where we present the status of drug shop operators.” (KII, Inspector, Luuka district)

“The sector has no much funds allocated for inspection. So, what we do is that normally during licensing, the drug inspector goes down to the ground to inspect. Occasionally, when members of the district health staff go out for integrated support supervision is when we check on drug shops. This is done on our way to supervise health facilities and PNFPS. Basically, that’s the method we employ.” (KII, Inspector, Buyende district)

Both focus group discussions with nurses in the two participating districts said that inspection was delegated. Most pertinent in the discussions was that even though the DDIs who draw the inspection mandate from the office of the DHO were the right people to carry out inspection, many a time, the inspection was a team effort done in tandem with other district officials such as sub-county coordinators and health assistants visiting health facilities within the district.

“Another method[of inspection] is the use of health assistants both at district and sub county level. They come and inspect and guide us where we are not doing well.” (FGD, Nurses, Buyende district)

In most of the focus group discussions with nursing assistants, there was a general consensus that DDIs did most of the routine inspection. The FGDs revealed that the initial inspection of drug shops was carried out by the DDIs. Beyond this stage, participants said that DDIs did not inspect them as stipulated in the local governments’ recruitment guidelines for health workers [35]. As such, on days when the DDIs felt like it was time to inspect drug sellers, district officials other than themselves were asked to carry out inspection visits. As far as frequency of visits were concerned, seven out of ten FGDs revealed that DDIs inspected every 3 months while the other three FGDs reported that DDIs were inspected every 4 months.

“They have been sending chairpersons to come and inform us down there [at drug shop premises] but the inspector has not been visiting some places although he calls us. Some of our colleagues got an opportunity and he visited them but some of us he has not come yet. He told us he will come because he has transport, that he will move and get to us.” (FGD, Nursing assistants, Buyende district)

### Licensing

All inspectors and members in FGDs intimated that during the inspection, the very first items drug inspectors ask for from drug sellers were their qualifications and a license. Inspectors said that the biggest hindrance in acquiring licenses by drug sellers was the lack of qualifications. The inspectors said that many drug sellers fell short of the requirements hence operated illegally. To help the drug sellers overcome this hurdle, attempts at getting them qualified people also referred to as ‘cover supervisors’ were made. Nonetheless, the drug sellers did not have enough money to pay cover supervisors for their academic transcripts which are a requirement for registration ultimately hampering the registration process. The quotes below are an illustration of what was said by inspectors.

“..of course, we inspect to know whether the premises are still suitable because you know these are drugs. If the premises are not suitable, they[drugs] can expire. If there is too much heat, they are exposed to sunlight. To make sure that the expired drugs are segregated, we look at records like sales records and where they[drug sellers] buy the drugs from. Of course, they[inspectors] also check on the personnel to see if the person they gave the license is still the person at the premises. So, that is now the routine surveillance. And also maybe to find out that they have the right drugs which are approved for drug shops. So briefly, that is what we do all the time, the year-round.” (KII, Inspector, NDA)

“What I have discovered is that it is not their[drug sellers] own making but lack of qualifications to get a license. Secondly, most of them lack finances. We used to recommend to them to get a cover-up person so that they run the drug shops but they can’t meet the expense involved.” (KII, Inspector, Luuka district)

The focus group discussion with nurses in Buyende district revealed that the kind of drugs sold vis-à-vis what the guidelines accepted posed a licensing problem. The nurses intimated that they had always been told by the DDIs to sell only drugs in class C and not A or B. Therefore, many inspection visits to premises owned by nurses were aimed at enforcing the right class of drugs. On a positive note, the nurses said that DDIs discouraged them from selling government drugs in their retail outlets. Rather, the nurses were encouraged to have referral books, stock rapid diagnostic kits, anti-malaria drugs, dispersible amoxicillin tablets, and oral rehydration salts—all used in the treatment of childhood febrile illnesses. Additionally, inspectors checked whether premises had a ceiling.

“They[inspectors] check on the way we keep our drugs in the shops, they check on the way we do and manage our activities. They look up to see whether there is a ceiling[in the operating premises].” (FGD, Nurses, Buyende district)

The majority of the focus group discussions with nursing assistants revealed that they were encouraged by DDIs to follow the right procedure in acquiring licenses allowing them to operate drug shops as mandated by the law. In two-thirds of the focus groups, drug sellers felt discouraged at getting licenses because whereas some of them struggled with a very strict process and eventually got licensed, some other drug sellers continued operating without one, and yet no punitive measures were taken. They said this created an atmosphere of unfair competition since many drug sellers had to part with huge sums of money to pay people with the right academic qualifications needed for licensing. The unlicensed drug sellers preferred to close their drug shops when they knew there was an ongoing round of inspection.

“When inspectors from NDA are moving around, those without a license immediately close[their drug shops], and for you, in that case, you remain open because you are on the right track. Even the patients get to know that you are the right person and strike a new friendship with you.” (FGD, Nursing assistants, Buyende district)

### Training

One of the two FGDs with nurses revealed that participants had gained a lot from the inspection visits. Members revealed that inspectors had taught them how to organize their drug shops in a much better way. These views are outlined below.

“We have benefited so much from the different trainings. Some of us didn’t know how to organize our drug shops and proper malaria treatment so we have learned a lot.” (FGD, Nurses, Buyende district)

In two-thirds of the focus group discussions with nursing assistants, drug sellers said that district drug inspectors informed them when to stop using certain drugs when a new drug was introduced. This also applied to change in technology such as the use of rapid diagnostic tests in the treatment of malaria. The nursing assistants also talked about the advice they received in terms of change in dosage that should be administered to patients besides stocking the right class of drugs.

“So, in this new way of working, we get updates well. When the inspectors come and find there is something new, they inform you that technology has changed. We didn’t know RDTs but because of inspection, we were able to know them. I am not sure of my colleagues’ opinion, but I think it[inspection]has done very well.” (FGD, Nursing assistants, Buyende)

### Bribes

What seemed glaring in all the FGDs was the fact that the current method of inspection is rife with illicit solicitation of money from drug sellers. During discussions, drug sellers told investigators of the study that DDIs ask for money from them with the intention to use the money for buying fuel for motorcycles used during the inspection process. Other members in the FGDs said that the actual intention of the inspectors was not inspection per se but rather getting money from drug sellers for personal purposes. What was most disheartening was that the money was asked for in an intimidating manner.

“Their intention of coming is not to inspect and teach us. They just come to make money. Whatever they ask you, it is like they are intimidating you but not to teach and help you to learn.” (FGD, Nursing assistants, Buyende district)

“..during their inspection, they[inspectors] usually ask for fuel money. It is quite common.” (FGD, Nursing assistants, Buyende district)

### Challenges of the inspection process

Key informants from the MoH and NDA said that the meager salaries paid to inspectors were partly to blame for the flawed inspection. They noted that there was a need to enhance salaries at all levels of inspection in order to improve the working conditions associated with the inspection of the private sector. In addition, the key informant from the NDA said that the lack of adequately trained drug sellers was a huge challenge.

“It is a big challenge to attract those[trained] people to the hard to reach areas. What happens is that you find the people who are qualified are people who are working in health centers. Sometimes, those very people are the ones who have been licensed. So, sometimes, when this[trained] person is working[at the health center], they are supposed to close the drug shop. However, sometimes they leave a nursing aid. Such things happen. That is one of the challenges we are always trying to solve.” (KII, Inspector, NDA)

At the district level, policy and drug shop challenges were talked about by the DDIs. They cited examples where they had gone for inspection but only found two or three drug shops open and willing to be inspected. This was because some drug sellers had failed to pay “cover supervisors” and as such, lacked licenses. The DDIs feared that this kind of behavior was not good as it compromised the quality of care provided. The DDIs also said that many drug sellers feared inspectors because the drug sellers had been handled in a very rough manner in the past. The DDIs attributed this rough handling to the lack of licenses which many drug sellers did not have. They reiterated the fact that even the meager amounts of money that were sent from the NDA to support the inspection process were not timely which greatly stifled routine inspection.

“In inspection, we have barriers entirely on our side and others by policy. Some times we can’t do frequent inspection because of limited resources. As for the drug sellers, they have the “I don't care” attitude. They open without “cover-up” or trained people, hence lowering the quality of service. On the policy side, we need more empowerment. We get supervisors from NDA to do the work[inspection] but it is not as frequent as it should be.” (KII, Inspector, Luuka district)

At the drug shop level, the majority of the FGDs with nursing assistants revealed that sometimes, the community was so poor to afford the recommended full dose. So, if the drug sellers insisted on selling drugs which the community could not afford, the clients went to the neighboring drug seller who was willing to sell to them an incomplete dose. In addition, on slight improvement, patients did not seek treatment from higher-level health facilities when referred because the health facilities were very far away.

“The community is poor. So if you think of giving them the recommended doses you won’t survive in business.” (FGD, Nursing assistants, Buyende district)

“The patients are also far from the government facilities and when they come to you for first aid and improve slightly, they don't go to the health facilities. So I request they allow us to sell those other drugs[prescription drugs] to help them recover.” (FGD, Nursing assistants, Luuka district)

## Discussion

The study set out to assess whether the current methods used to inspect drug sellers mirrored inspection guidelines as highlighted in the national professional guidelines for licensing, renewal, and new licenses for class C drug shops in Uganda. Study results revealed that the current methods of inspection were unpredictable, authoritarian, and characterized by illicit payments whenever they happened. Otherwise, inspection is a delegated process aimed at looking for drug sellers without valid licenses. Drug sellers felt there was a need to make the licensing process easier than it is currently. Drug sellers also felt there was a need for more training courses on childhood diseases as this would contribute to smooth running of the business.

The study found that drug sellers appreciated the training they received from drug inspectors in as far as dispensing drugs and treatment of patients were concerned. This was against a background that the national professional guidelines for licensing, renewal and new licenses for class C drug shops in Uganda do not provide for drug inspectors teaching drug sellers how to treat patients. Drug sellers complained about inspectors asking for illicit money in the form of bribes. The challenges of inspection were mainly to do with inadequate funds and personnel who carry out inspection.

While the national professional guidelines for licensing, renewal and new licenses for class C drug shops in Uganda where inspection is enshrined implicitly prescribes cordial inspection, the current method of inspection is authoritarian and is aimed at fault-finding with little or no room for negotiations similar to what has been reported elsewhere [8]. Studies reporting positive inspection outcomes to highlight the need for dialogue between inspectors and the people being inspected [42]. In Uganda, every district has one DHO and one DDI who are the statutorily recognized inspectors. This two-man workforce is supposed to inspect many drug shops spread over a wide geographic area in the district. It is important that this two-man workforce together with the national inspectors improvise catalytic actions based on dialogue and suggestions between themselves and drug sellers as a way of improving compliance to inspection. This finding concurs with a study that examined the use of authority in enforcing compliance of ward leaders. Through catalytic actions based on dialogue and suggestions, enforcing compliance increased the knowledge of and compliance with rules among motivated ward leaders [43]. This approach may improve compliance with drug sellers if adopted by inspectors in Uganda.

The current method of inspecting drug sellers is characterized by impromptu raids aimed at surprising drug sellers similar to what has been documented in some Asian countries [8, 44] and yet, one of the functions of inspectors of private drug shops in Uganda is to make a continuous review of the needs, knowledge, and resources of drug sellers which does not happen in practice. The surprise visits are usually synergized with law enforcement whose sole purpose is to arrest drug sellers found selling drugs that are not permitted by law. As such, inspectors are accompanied by law enforcement agencies such as the police because they are duty-bound by the mission of the NDA which is to ensure the availability at all times of essential, efficacious, and cost-effective drugs to the entire population of Uganda. Therefore, the mission and function of inspection have to be achieved regardless of what the drug sellers feel because illicit trade is prohibited by law.

According to the regulatory theory, policy intent and enforcement practices need to be interpreted within context. The main assumption of the current national professional guidelines for licensing, renewal, and new licenses for class C drug shops in Uganda is that once drug shops are licensed, drug sellers ought to engage in the right practices. Therefore, the intention of the current policy is to enforce compliance even if it means involving the police. However, several studies have reported that licensed drug sellers engage in selling prescription drugs and yet they are permitted to sell over the counter drugs only [45, 46]. For as long as drug sellers are not able to see the financial benefit accruing from adhering to policy guidelines and high handed punitive measures persist, it is more likely that they will continue to engage in practices that contravene the law.

The manner in which inspection of drug shops is currently carried out in Uganda is indicative of a glaring gap in the inspection structure. This is because practically, the inspection can be done by anybody ranging from a sub-county coordinator, councilor, parish chief, district environmental officer to an official from the Ministry of Health. This clearly contravenes the law since the DDI is the lawfully designated inspector for drug shops at the district level in Uganda. Elsewhere, it has been suggested that leadership in health care should prioritize patient care outcomes rather than structures and processes used to deliver health care. The ultimate target should be to deliver an effective service and not merely have an effective operation [45, 47]. In Uganda, the inspection is neither effective nor efficient because drug sellers close shop during inspection visits and as such inappropriate treatment of patients persists. There is a need for government inspectors to engage in more humane ways of inspection so as to reach every drug seller. Short of that, there will be elevated anti-microbial and anti-malarial resistance as a result of continued inappropriate treatment in children under 5 years

In other instances, DDIs gather drug sellers in designated places near their places of work and either pass on urgent information emanating from the NDA or caution them based on information gathered from impromptu visits of randomly visited drug shops. Whereas this method ensures that one of the tasks of inspection as stipulated by the law (mentoring and coaching drug shop owners about NDA new guidelines including treatment guidelines) is fulfilled, a lack of regular follow up due to shortage of funds is usually demotivating. It is therefore not surprising that inspectors compensate for this shortage of funds by soliciting illicit money in the form of bribes. These results agree with similar studies dealing with regulation of the private sector which found that regulation by actors may be hindered by organizational challenges which may not be limited to lack of motivation as a result of inadequate incentives [3, 48]. There is a need to identify recurrent funding, especially in low-income countries if inspection especially of retail drug shops is to be sustainable.

Whereas the law stipulates a pharmacy technician as the most suitable inspector for drug shops, this is not what happens in practice. In many parts of the country, the DDI is either a clinical officer or a nurse. This is mainly due to a shortage of qualified personnel and has even led to task shifting in some contexts as has been documented in studies done elsewhere [9, 49]. As a result, the NDA has set up regional offices manned by pharmacists also referred to as zonal inspectors. The zonal inspectors are re-trained by officials from NDA on how to carry out inspection. Nonetheless, the zonal inspectors have also been described as being high handed and too authoritarian in the way they carry out inspection. Again, from the regulatory theory, we postulate that drug sellers may comply rationally to set guidelines motivated entirely by personal benefit or they may obey morally when authorities use procedural fairness because of an intrinsic obligation to do the right thing [50]. It is therefore important to note that even though the MoH, NDA, and the districts add more layers of inspectors, unless drug sellers understand what they benefit from the inspection process, compliance will remain an uphill task. Only drug sellers who feel that inspection helps to legitimize their trade and validate them among the communities they serve will embrace inspection visits.

Relatedly, drug sellers complained about being embarrassed mostly by zonal inspectors and sometimes by DDIs in front of patients being treated. This stigmatization often results in disapproval when directed at drug sellers and their behavior. When the drug sellers are treated disrespectfully with no room to amend deviant behavior, then deviance becomes a master status trait as explained by studies done elsewhere [51, 52]. That is why during inspection visits, drug sellers close shops in a bid to avoid being embarrassed and could be one of the reasons why inappropriate treatment practices persist. Conversely, in a few instances, drug sellers reported that the current methods of inspection improved their performance. Even then, the performance mentioned was to do with compliance with cleanliness and general set up of premises which is the intention of the current guidelines. Studies done elsewhere also concur that the ultimate aim of the inspection is to improve compliance [6].

Studies show that delegated inspection may not lead to compliance because of its temporary nature which more often than not leads to tension between inspectors and healthcare providers [49]. In Ugandan rural settings, whenever the DDI and zonal inspectors are not able to make it for routine visits, inspection is delegated to proxies such as members of the DHT or other actors at lower village levels such as councilors and sub-county chiefs. These proxy inspectors often lack professional experience to perform a professional role. To the proxy inspectors, what is of utmost importance is power over drug sellers which has been associated with dismay. This is because the proxy inspectors create unnecessary tension and worry among drug sellers and yet drug sellers do not feel like they get much intellectual help from the proxy inspectors. This impasse has been overcome in the public sector with the human resource department of the ministry of health ensuring that there is adherence to set standards for effective delivery of the National Minimum Health Care Package (UNMHCP) by determining personnel needs both in numbers and skills as may be required by the national health policy [53]. Post-graduate and in-service training aimed at improving skills and technical competencies; quality performance and productivity of the health workforce are also mandates of the human resource department. In essence, the department is charged with the inspection of human resources in order to make sure that set standards are adhered to.

On the other hand, the quality assurance department of the Ministry of Health is charged with the supervision of the public sector [54]. To this effect, supervision guidelines have been drawn and effected [55]. The other department in the ministry of health is the pharmacy department whose mandate of regulating the private sector is executed by the NDA [56].

It is, therefore, puzzling why the ministry of health regulates the public sector using standard structures and guidelines as far as inspection and supervision is concerned and only engages in inspection with no formal support supervision for the private sector. Moreover, whereas there is a dearth in studies showing how inspection translates into quality health care [6], there is a plethora of information pointing to the effectiveness of supervision on the quality of health care [57, 58]. It is, therefore, important that the ministry of health together with the NDA initiate formal support supervision for drug shops since regulatory inspection alone has not been effective in improving the quality of health care offered by private drug sellers.

The major strength of our study is that it builds on the perspectives of inspectors and drug sellers in highlighting the difference in how the inspection is carried out in relation to how it should be carried out. This is further strengthened by the use of theories such as the regulatory theory in discussing some of the results that would otherwise be abstract.

A major limitation of this study is that it only considered licensed drug shops yet unlicensed ones also exist. The investigators of the study are aware that unlicensed drug shops also provide health services to communities in which they are located and would have provided findings that are more representative of the general situation in the study area. However, the investigators wanted to get a good understanding of regulatory inspection from drug sellers who operate legally and are recognized by the law hence the decision to omit the unlicensed drug shops. Findings from this study provide sustainable solutions to a private sector that plays a pivotal role in health service provision in Uganda.

## Conclusion

The current method of inspecting drug sellers is harsh and instills fear among drug sellers. There is a need to establish channels of dialogue between inspectors and drug sellers if meaningful compliance to inspection and adherence to policy guidelines is to be achieved. The government needs to enhance both human and financial resources if meaningful inspection of drug sellers is to take place.

## Data Availability

Datasets used during the study are available from the corresponding author on reasonable request.

## Abbreviations

DDI: District drug inspector;
DHO: District health officer
FGD: Focus group discussion
IDI: In-depth interview
NDA: National Drug Authority
PNFP: Private not-for-profit
